# Scientific research input and output efficiency evaluation of universities in Chengdu–Chongqing economic circle based on data envelopment analysis

**DOI:** 10.1371/journal.pone.0287692

**Published:** 2023-07-07

**Authors:** Chong Wang, Jun Zeng, Hui Zhong, Wei Si

**Affiliations:** 1 School of Economics and Management, Sichuan Normal University, Chengdu, Sichuan, China; 2 School of Tourism Culture Industry, Sichuan Tourism University, Chengdu, Sichuan, China; Hebei Agricultural University, CHINA

## Abstract

The study takes 10 urban agglomerations in China as the research object, focusing on the Chengdu-Chongqing urban agglomeration, and applies Data Envelopment Analysis (DEA) to measure and compare their scientific input and output efficiency of universities. First, this paper analyzes the input and output of scientific research in universities in major provinces in China in detail. Second, according to the construction principles of the indicator system, using qualitative interview to construct evaluation indicators of university research efficiency. Third, using DEA method, first analyze the input and output profile of some urban agglomeration universities such as Chengdu-Chongqing economic circle, measure and compare their research input and output efficiency, then compare and analyze the research efficiency of research-type sample universities within Chengdu-Chongqing economic circle, and conduct a projection study of non-DEA effective sample universities. The main conclusions are as follows: first, the average efficiency of scientific research in universities in Chengdu-Chongqing and other urban agglomerations in 2020 has slightly increased compared with that in 2016, but the gap between urban agglomerations is prominent, and the innovation level of scientific research in higher education institutions in urban agglomerations needs to be improved. Second, there is a mismatch between the themes of research, funding and human resources in research-oriented universities in the Chengdu-Chongqing economic circle. Third, there is considerable room for improvement in research efficiency, and the influence of scale on overall efficiency is weak. We found that excessive investment in scientific research in universities is the main reason for the non-effect.

## Introduction

Technological innovation depends on the input of scientific research. Universities have abundant scientific research resources and high-quality scientific research personnel, and take on the vast majority of scientific research projects, so universities are an important component of input in scientific research. The efficiency of regional innovation is the efficiency of the use of investment in regional scientific research. Improving the efficiency of regional innovation means that the same investment in scientific research can produce a greater output, or that the same output of scientific research requires less investment. As the leaders of regional innovation, universities in urban agglomerations have a direct impact on the development and coordinated operation of urban agglomerations. Some scholars have studied the regional differences in the efficiency of scientific research in colleges and universities. A recent study has conducted an empirical analysis of the efficiency of 32 world-class universities by using the DEA-fsQCA model, and shows that there are regional differences in the efficiency of university research [1].

What is the efficiency of scientific research in the Chengdu-Chongqing economic circle, the most innovative economic region in western China? Does the large input of resources match the output, and are the resources maximally utilized? Understanding the use of resources for scientific research in universities in the Chengdu-Chongqing economic circle can help improve the innovation capacity of scientific research, build a high-quality education system, and optimize the allocation of local higher education resources. The research results of universities are the key factors for economic growth, so evaluating their efficiency is an important aspect.

Two American operations research scientists proposed the DEA method in 1978, but it was not until the 1990s that it began to be used in educational evaluation. Scholars have done some research on models of evaluating the efficiency of scientific research in colleges and universities. The methods of evaluating the efficiency of scientific research in colleges and universities mainly include the Cobb-Douglas production function method, comprehensive input-output analysis, comprehensive fuzzy evaluation, the Malmquist index, data envelopment analysis, and stochastic boundary analysis. Most researchers have used the DEA to analyze the efficiency of the input to and output of education, and the relevant works can be divided into three categories according to the research objects considered. **Macroscopic view**: This involves looking at colleges and universities in different regions. Some researchers used the output-oriented, weight-constrained BCC window analysis model to calculate the average total factor productivity of 32 national double first–class universities from 2015 to 2018 [2]. A multi-criteria decision making (MCDM), non-radial, super-efficient data package model of network analysis was proposed to show the differences between disciplines and distinguish the evaluation results. Then, they used the Malmquist index to decompose the changes in the efficiency of productivity of research in statistics departments in different universities in China [3]. DEA and spatial measurement methods were used to discuss the temporal and spatial patterns of research efficiency in 31 provinces from 2009 to 2019, and found that their efficiency exhibited the Matthew effect [4]. **Middle view**: This research considers a certain type of university or a single university. Some researchers considered 15 universities in Hebei Province from 2016 to 2018, used the DEA model to evaluate the overall efficiency of each university’s scientific research input and output, and used the Malmquist index to analyze the dynamic change of efficiency [5]. Based on factor analysis, a statistical analysis of the efficiency of scientific research of five types of colleges and universities was conducted: comprehensive, engineering, agriculture and forestry, medicine and normal education. Study presented some decision units are mixed when measuring efficiency by using standard DEA models, so they may belong to multiple groups, and the total number of observations is limited [6]. A study applied a three-stage DEA and the Malmquist index method to evaluate the static and dynamic efficiency of input–output data of scientific research produced by universities directly under the Ministry of Education in the period of 2010 to 2017 [7]. **Microscopic view**: This involves looking at the internal departments or disciplines of a particular university. Some researchers prefer to use the DEA model to collect data on scientific research from colleges of humanities and social sciences to build a super-efficiency model, and the results showed significant differences in their efficiency [8]. In addition, some researchers analyzed expert opinions on the factors influencing scientific research capability in 13 colleges and universities, extracted weights by using the analytical hierarchy process, and established seven factors of a system for evaluating scientific research in colleges and universities [9].

Scholars have also conducted relevant research on the evaluation indicators of scientific research efficiency in colleges and universities. The first to study the input and output of scientific research in Chinese universities used the available funds and personnel as the main input indicators, and used talent training and published academic literature as the main output indicators to measure the efficiency of scientific research [10]. There are several measurement indexes of the input and output of scientific research. A first measurement index is that used the number of teachers and researchers, the number of graduate students, the number of federal grants, and the size of library collections as indicators of input in research [11], and one study also explored the efficiency of scientific research in seven academic institutions in the U.S., and used the number of faculty members and financial resources in these institutes as input indicators [12]. A second measurement index is that which used the number of faculty members, operating cost, and floor area as the input factors, and used the total number of teaching hours, number of dissertations, and external grants as the output factors [13,14]. In addition, a study used the number of people employed and the funds allocated to universities in a given year as the input indicators, and the number of scientific and technological achievements at the provincial and ministerial level or higher, and the number of publications and patent authorizations as the output indicators [15]. A study compared the differences among 24 medical colleges and universities in four provinces over 10 years. The selected input indicators were the research and development personnel and the scientific and technological funds; the output indicators were the number of monographs and scientific papers published in foreign and domestic journals, the number of evaluation results, the income from technology transfer, the number of awards, and the citations in publications from the Web of Science core collection [16]. Some studies used manpower, and scientific research funds and projects as the input, and used technology transfer, and scientific and technological achievements and awards as measures of output [17,18].

Based on the current literature, most scholars still use the DEA method to study the efficiency of scientific research in universities [19]. At the same time, scholars have also established the evaluation index system of university scientific research input and output, and used these indicators in the research of university scientific efficiency. Generally, human and financial resources are used as the main input indicators, and the number of published papers and patents and income are used as the main output indicators. But there are certain differences in the selection of evaluation indicators used by experts and scholars to study the input and output of scientific research in colleges and universities, and the subtle differences in the selection of indicators are mainly reflected in secondary and tertiary indicators.

However, there are shortcomings in the existing research. First, a system of indices to assess the input and output of universities has not been developed for China. Second, the sample data selected by researchers are generally relatively old, which hinders a sound understanding of the latest scientific research in Chinese universities. Third, scholars have generally focused on internal departments in universities, certain types of universities, and universities in different regions of the country. Few scholars have analyzed the efficiency of scientific research in colleges and universities in different urban areas, which may reflect regional innovation capabilities.

In summary, the contributions of this paper are mainly: (1) We expand the scope and perspective of the study, and used a data of scientific research input-output from 10 urban agglomerations in China to measure the efficiency of input-output. (2) Select DEA-BCC method to analyze the static efficiency of scientific research of universities, and used the Malmquist index to analyze the dynamic effects of scientific research of universities. (3) We build the input and output indicators of university scientific research through literature analysis and qualitative interview, this paper improves and completes the existing evaluation index system of scientific research input-output efficiency. In addition, we used the constructed indicator system to analyze the input-output efficiency of university scientific research in Chengdu-Chongqing economic circle, which also makes up for the limitations of the current study.

## Materials and methods

### Methodology of DEA

The reason for choosing DEA in this paper is that DEA is applicable to the evaluation of scientific research efficiency in universities. First, due to DEA’s wide applicability, simple principle, and unique advantages especially in analyzing multiple inputs and outputs, it has been widely applied to the performance evaluation of various organizations and regions, such as banks, enterprises, governments, research institutions, hospitals, and other fields as a non-parametric estimation method for evaluating the relative effectiveness of research objects [20]. Second, using DEA can not only comprehensively evaluate the input-output of scientific research in universities and obtain comprehensive efficiency, but also calculate the technical efficiency and scale efficiency of each decision-making unit. It can also find out the reasons why each decision-making unit is not DEA effective through projection analysis [21]. Third, DEA is suitable for the comprehensive evaluation of the efficiency of multi input and multi output activities. It combines several mathematical programming such as linear programming, and has a unique applicability for the evaluation of input–output efficiency in universities. At present, there are many applications of DEA methods in multi input and multi output activities at home and abroad, especially in the evaluation of higher education [22]. Therefore, the use of DEA method to evaluate the efficiency of scientific research in Chinese universities is appropriate.

DEA model occupies the main position in the evaluation method because of its uniqueness of "evaluation" and "input and output" of scientific research [19,23]. The American operations research scientists proposed the DEA method in 1978 [24]. It is a method of quantitative analysis used to evaluate the relative effectiveness of the same type of units based on multiple input and output indicators. The basic idea of the DEA method is to take each evaluated unit as a decision-making unit (DMU, for short) or department, comprehensively analyze its input-output ratio, and weight and use it as a variable for calculation. In this way, the effective production frontier can be determined. The distance between each DMU and the effective production frontier is used to determine its validity for DEA. The projection method is used to identify the reasons for the inefficiency of non-DEA-effective DMUs and suggest directions for improvement [25–27]. DEA has two models, one is the Charnes, Cooper, and Rhodes model (CCR), the other is the Banker, Charnes, and Cooper (BCC) model [28]. The CCR model is the simplest DEA model, which assumes constant returns to scale. That is, assuming that the DMU scales-up its output by proportionally increasing its input, the size of the DMU does not affect its efficiency [23,28]. The BCC model is an improvement of the CCR model [29]. It assumes that scale returns change, and decomposes technical efficiency (TE) into efficiency of scale (SE) and pure technical efficiency (PTE), that is, TE = PTE*SE. PTE is obtained from the objective function, and according to SE = TE/PTE, it gives a scale-efficient SE. Therefore, the BCC model is as follows:

$$\begin{array}{c} \text{min}\theta \\ s.t.\sum_{j=1}^{t}\lambda_{j}x_{j}\leq \theta x_{0},\\ \sum_{j=1}^{t}\lambda_{j}y_{j}\geq y_{0},\\ I\lambda =1\\ \lambda_{j}\geq 0,j=1,2,\ldots ,t. \end{array}$$

where I = (1,1,…, 1)_1 * t_.

Pure technical efficiency (PTE) is obtained by an objective function and scale efficiency (SE) is obtained by SE = TE / PTE. Based on the indicator values, we can evaluate the overall input-output efficiency of scientific research in colleges and universities.

#### Malmquist Index

In order to reflect the dynamic efficiency, we will use Malmquist index for data analysis. This model has been used for dynamic measurement of universities scientific research efficiency [4]. The Malmquist index is based on the DEA method, and is also known as the total factor productivity index. The Malmquist index was proposed in 1953 by the Swedish economist Malmquist [30]. The index was used in 1982 to measure changes in productive efficiency. Researchers have combined DEA with a nonparametric method of linear programming for this theory, and it has been widely used since then. It is mainly used to observe changes in the efficiency of DMUs in a given period and can be decomposed into technical progress (TE) and technical efficiency (EF). EF can be divided into scale efficiency (SE) and pure technical efficiency (PE) [31]. It is expressed as follows:

$$Z_{0}\left(m^{i+1},n^{i+1},m^{i},n^{i}\right)={\left[\frac{D^{i}\left(m^{i+1},n^{i+1}\right)}{D^{i}\left(m^{i},n^{i}\right)}\times \frac{D^{i+1}\left(m^{i+1},n^{i+1}\right)}{D^{i+1}\left(m^{i},n^{i}\right)}\right]}^{\frac{1}{2}}$$ (1)

The distance function in period i is represented by D^i^, the input value in that period is represented by m^i^, and the output is represented by n^i^. If total factor productivity declines over time, Z_0_ is less than one, and is equal to one if the total factor productivity does not change over time. If the productivity increases over time, Z_0_ is greater than one. TE reflects the organizational management of the DMU, and technological progress reflects changes in the level of innovation of the DMU. It can be used to measure the efficiency of scientific research, organizational management, and the level of innovation in colleges and universities.

We selected DEA-BCC model and Malmquist index, and conducted a static and dynamic analysis of the input-output statistical data of universities in Chengdu- Chongqing economic circle from 2016 to 2020. The traditional DEA models are the BCC model and the CCR model, but the CCR model emphasizes that fixed returns to scale remain unchanged. The BCC model introduces variable returns to scale (VRS) based on the CCR model to measure pure technology and scale efficiency [26,27]. This study mainly analyzes the actual behavior of the impact of scientific research input on the overall factors of scientific research efficiency management. Therefore, we selected the BCC model. In addition, the BCC model is a static analysis method to evaluate the relative efficiency of university research efficiency, but it cannot vertically compare the efficiency values of different universities. Therefore, this paper also selected the Malmquist index, which is used for a dynamic analysis [27].

### Index selection

#### Selection of variables

The scientific research conducted in colleges and universities is complex and diverse. Although a set of standardized indicators of the efficiency of this research has not yet been formulated, the general direction is clear. First, we review and summarize the results of academic research on the input and output of scientific research through a literature review, formulate a system of indices based on it, and then categorize and summarize it as the initial basis for the selection of the input and output indicators. Second, we use qualitative interview with laboratory management experts and scientific researchers to repeatedly adjust the index system. Finally, we use the rules of decision-making units (DMUs) and the evaluation indicators of the DEA method to formulate the final indicators for evaluating the efficiency of scientific research in colleges and universities.

First, we searched the relevant literature by using the keywords "scientific research efficiency evaluation", "input and output of colleges and universities", and "scientific research evaluation of urban agglomeration colleges and universities". Combined with the journal impact factor and download volume, a total of 32 literatures with high reference value such as Peking University Core and Chinese Social Sciences Citation Index (CSSCI), were obtained. Then, we extracted the indicators (including through repetition and crossover) that had been used in the selected studies. The indicators "investment by government departments", "investment by enterprises and institutions", "number of international academic conference participants", and "area of premises" appeared in less than 7% of the 32 studies, which was significantly lower than the frequency of the other indicators. The design perspectives of the evaluation indicators in the above studies may have been different, but they were highly representative. We selected the 15 most common terms as preliminary indicators, as shown in Table 1.

**Table 1:** Preliminary indices selected based on literature review.

	Index	Frequency/%
1	Papers published in foreign journals	65.625%
2	Published papers in domestic journals	65.625%
3	Published work	59.375%
4	Full-time staff equivalent for research and development	53.125%
5	Number of patents granted	43.750%
6	Actual income from patent transfer	43.750%
7	Achievement awards	34.375%
8	Investment in research funds	31.250%
9	Internal expenditure for scientific research	28.125%
10	Senior teachers and research personnel	25.000%
11	Number of identification results	25.000%
12	Cumulative number of subjects	15.625%
13	Longitudinal research funding for the year	12.500%
14	Latitudinal research funding for the year	12.500%
15	Fixed assets acquisition fee	12.500%

Source: Literature review; compiled in Excel.

Then, we conducted offline interviews with laboratory management experts and scientific researchers to make the indicators more realistic and reflective of the input and output of scientific research in colleges and universities. The main contents of the interviews included the number of subjects taught at the time, the use of equipment and funds, the composition of research groups, the corresponding results and published articles, and the use of fixed laboratory assets. We sorted and summarized the content of the interviews, extracted the main input-output elements, and combined them with the preliminary indicators above to obtain a final set of indicators, as shown in Table 2.

**Table 2:** Indicators to assess efficiency of scientific research in colleges and universities.

Primary indicator	Secondary indicator	Tertiary indicator
Input indicators	Human resources	Senior teachers and research personnel (persons)
		Full-time staff equivalent for research and development (persons/year)
	Research funding	Research funds allocated for the year (thousands of yuan)
	Number of subjects	Cumulative number of subjects (items)
Output Indicators	Monograph Papers	Published work (department)
		Academic papers (articles)
	Social benefit	Income from technology transfer (thousands of yuan)
	Award-winning results	Achievement awards (items)

#### Selection criteria

From the literature review we concluded that the investment in scientific research in colleges and universities mainly included human, financial and information resources, the fixed assets consisted of experimental equipment, and the annual changes were small and difficult to calculate. Therefore, these indicators were included in the financial investment. The number of senior teaching and scientific research personnel, the equivalent of full-time research and development personnel, the allocated annual funds for scientific research, and the total number of projects were selected as input indicators. The output indicators consisted of the number of monographs, published papers, patents and awards for achievements.

"Research and development personnel" in this article refers to faculty members who spent more than 10% of their time on research and development during the year. "Full-time staff" refers to those who are engaged in research and development or research and application development during the statistical year, and whose scientific and technological service hours account for more than 90% of their total working hours (excluding winter and summer vacations and overtime hours, it is calculated as 10 months per year). The senior professional title is the highest level in the professional title, and it is divided into two categories: senior and vice-senior. The sources of scientific research funding included government grants, funds from companies and institutions, and some funds raised by universities/researchers themselves. The number of projects included vertical projects directly funded by scientific research grants from the national and provincial governments, and horizontal projects involving technical cooperation to obtain R&D funds. The quantity and quality of papers and monographs were not only the key output indicators for universities to enhance their competitiveness in scientific research, but also the simplest and most intuitive way to evaluate research. Patented technology is often used to assess the applied value of scientific research and can reflect its social and economic benefits. Approved patents indicated that the relevant scientific research had a certain applicative and developmental value, and the actual technology transfer income signified the income from the commercialization of the research. Achievement awards signified recognition of the quality of scientific research, and included awards at the national, provincial, and university levels.

## Results

### Data sources

The data used for this research were obtained from the Chinese government’s "Compilation of Science and Technology Statistics of Higher Education Institutions" and official websites, such as that of the Ministry of Education. In order to compare the differences in scientific research input output efficiency between universities in Chengdu-Chongqing economic circle and other urban agglomerations in China, we conducted an overall comparison between universities in the Chengdu-Chongqing economic circle and those in other urban agglomerations. Through the comparison, we hope to find out the level of scientific research input and output of universities in the Chengdu-Chongqing economic circle at nationwide. Since most colleges and universities are located in provincial capitals, data from the relevant provinces were used in the analysis [32].

### Comparative analysis of efficiency of scientific research in universities in China’s 10 densest urban agglomerations

According to the "Beijing-Tianjin-Hebei Metropolitan Circle Regional Planning ", "Pearl River Delta Region Reform and Development Plan Outline ","Yangtze River Delta Urban Agglomeration Development Plan "and "Chengdu-Chongqing Economic Circle Construction Plan ", the urban agglomerations were divided into geographically similar provinces and cities with complementary advantages in production [33,34]: the Beijing-Tianjin-Hebei urban agglomeration (Beijing, Tianjin, and Hebei), Yangtze River Delta urban agglomeration (Zhejiang, Jiangsu, Anhui, and Shanghai), Pearl River Delta urban agglomeration (Guangdong), the urban agglomeration in the middle reaches of the Yangtze River (Jiangxi, Hunan, and Hubei), Chengdu-Chongqing urban agglomeration (Sichuan and Chongqing), Central Plains urban agglomeration (Henan), Beibu Gulf urban agglomeration (Guangxi and Hainan), Guanzhong Plain urban agglomeration (Shaanxi), West Strait urban agglomeration (Fujian), and Harbin-Changzhou urban agglomeration (Jilin and Heilongjiang).

#### Overview of inputs and outputs

As shown in Table 3, universities in the Beijing-Tianjin-Hebei, middle reaches of the Yangtze River, Yangtze River Delta, and Pearl River Delta urban agglomerations were at the forefront in terms of absolute investment, and attained large inputs and outputs. Universities in the Chengdu-Chongqing and the Guanzhong urban agglomerations had small inputs and large outputs. Universities in the Central Plains and Beibu Gulf urban agglomerations had relatively low levels of inputs and outputs.

**Table 3:** Inputs and outputs of scientific research by colleges and universities in China’s top 10 urban agglomerations in 2020.

Urban agglomeration	Province	Senior teaching and research personnel (persons)	Full-time staff equivalent for research and development (persons/year)	Research funds allocated for the year (thousands of yuan)	Cumulative number of subjects (items)	Published works (department)	Academic paper (articles)	Actual income from technology transfer (thousands of yuan)	Achievement awards (items)
Beijing-Tianjin-Hebei urban agglomeration	Beijing	29167	32749	25368895	63698	625	99197	341293	371
	Tianjin	8229	9298	4145777	14312	84	25389	9355	135
	Hebei	16822	8696	2407844	12564	133	26745	10976	149
Yangtze River Delta urban agglomeration	Shanghai	20108	24078	14986233	38767	347	79040	158687	279
	Jiangsu	32736	23493	17356174	50899	369	110356	441038	499
	Zhejiang	18585	14447	9162636	36800	197	40016	144800	237
	Anhui	12607	11552	6853140	18446	170	30404	29882	239
Pearl River Delta City Cluster	Guangdong	25930	22583	15842487	48910	274	80631	177626	192
Urban agglomeration in the middle reaches of the Yangtze River	Hubei	22989	13605	10012318	30304	314	60395	132677	360
	Hunan	17006	14314	4919778	17684	194	44780	81309	237
	Jiangxi	10869	5949	2090519	11043	130	16836	28691	107
Chengdu-Chongqing urban agglomeration	Sichuan	18453	16218	6330685	32893	332	59355	312617	219
	Chongqing	9090	7309	3606782	14750	186	26162	240711	131
Central Plains city cluster	Henan	17073	6799	2729714	13842	451	35418	60602	236
Harbin-Changzhou city cluster	Jilin	12570	13474	3081642	11390	120	25965	95179	191
	Heilongjiang	16383	12674	5766399	14437	197	38708	25711	253
Guanzhong Plain City Cluster	Shanxi	18839	11225	9243642	35337	342	62171	183108	282
City group on the west coast of the strait	Fujian	10102	9994	4076270	19592	79	18687	23679	141
Beibu Gulf city group	Guangxi	8707	9080	2051913	14244	83	15967	13035	82
	Hainan	1786	667	272913	1421	29	2637	1822	14

Source: 2020 "Compilation of Science and Technology Statistics of Higher Education Institutions".

#### Analysis of overall efficiency

We used DEAP2.1 software to evaluate the efficiency of the inputs and outputs of scientific research by universities in the 10 densest urban agglomerations in China. Only the relevant data from 2016 and 2020 were compared and analyzed, and the results are shown in Table 4.

**Table 4:** Efficiency of the inputs and outputs of scientific research in universities in China’s 10 densest urban agglomerations in 2016 and 2020.

Urban agglomeration	Province	2016				2020			
		Overall Efficiency	Pure Technical Efficiency	Scale Efficiency		Overall Efficiency	Pure Technical Efficiency	Scale Efficiency	
Beijing-Tianjin-Hebei urban agglomeration	Beijing	1.000	1.000	1.000	-	1.000	1.000	1.000	-
	Tianjin	0.812	0.846	0.960	irs	1.000	1.000	1.000	-
	Hebei	1.000	1.000	1.000	-	0.856	0.864	0.991	irs
Yangtze River Delta urban agglomeration	Shanghai	1.000	1.000	1.000	-	1.000	1.000	1.000	-
	Jiangsu	1.000	1.000	1.000	-	1.000	1.000	1.000	-
	Zhejiang	0.795	0.833	0.954	irs	0.770	0.771	0.998	drs
	Anhui	0.752	0.770	0.977	irs	1.000	1.000	1.000	-
Pearl River Delta City Cluster	Guangdong	0.872	0.893	0.977	drs	0.867	0.871	0.995	drs
Urban agglomeration in the middle reaches of the Yangtze River	Hubei	1.000	1.000	1.000	-	1.000	1.000	1.000	-
	Hunan	0.978	0.978	1.000	-	1.000	1.000	1.000	-
	Jiangxi	0.877	0.905	0.970	irs	0.706	0.738	0.957	irs
Chengdu-Chongqing urban agglomeration	Sichuan	0.771	0.838	0.920	drs	1.000	1.000	1.000	-
	Chongqing	1.000	1.000	1.000	-	1.000	1.000	1.000	-
Central Plains city cluster	Henan	1.000	1.000	1.000	-	1.000	1.000	1.000	-
Harbin-Changzhou city cluster	Jilin	1.000	1.000	1.000	-	1.000	1.000	1.000	-
	Heilongjiang	0.862	0.864	0.998	irs	1.000	1.000	1.000	-
Guanzhong Plain city cluster	Shanxi	1.000	1.000	1.000	-	1.000	1.000	1.000	-
City group on the west coast of the strait	Fujian	0.722	0.759	0.950	irs	0.815	0.836	0.974	irs
Beibu Gulf city group	Guangxi	0.825	0.851	0.969	irs	0.722	0.768	0.940	irs
	Hainan	0.900	1.000	0.900	irs	0.758	1.000	0.758	irs
Mean		0.908	0.927	0.979		0.925	0.942	0.981	

“Irs” means increasing returns to scale; “drs” means diminishing returns to scale.

Source: Results of DEAP2.1 software.

Table 4 shows that in 2020, the average values of pure technology, scale, and comprehensive efficiency of scientific research in universities in Chengdu-Chongqing and other urban agglomerations increased by 0.015, 0.002, and 0.017, respectively, compared with those in 2016. The overall level of scientific research in universities in these urban agglomerations improved slightly. However, only a few urban agglomerations were in a DEA-effective state, and the gap in efficiency between them was large.

The comprehensive efficiency of colleges and universities in various urban agglomerations in 2020 increased by 1.9% compared with that in 2016. In 2016, only the Central Plains and Guanzhong Plain urban agglomerations effectively allocated resources for scientific research, but in 2020, the Chengdu-Chongqing and Harbin-Chongqing urban agglomerations had become comparably efficient with them. This indicates that the rate of utilization of resources for scientific research in universities in the western and northeastern regions has increased significantly. Although an increasing number of urban agglomerations are becoming effective according to DEA, the efficiency of scientific research in their universities needs to be improved, especially in the central and western urban agglomerations.

The pure technical efficiency of universities in different urban agglomerations in 2020 increased by 1.6% compared to 2016. In 2016, two urban agglomerations had reached an effective, indicating that their innovation, management of scientific research, and methods to assess performance were all satisfactory. In 2020, Tianjin, Anhui, Hunan, Sichuan, and Heilongjiang joined the list of effective provinces, and are located in the central, western, and northeastern regions of China. Hebei became a non-DEA-effective province, indicating that the pure technical efficiency of its colleges and universities had decreased.

The efficiency of scale of colleges and universities in various urban agglomerations in 2020 increased by 0.2% compared with that in 2016. Urban agglomerations that reached an effective state of DEA included Chengdu-Chongqing, Zhongyuan, Ha-Chang, and Guanzhong, and the efficiency of scale of the rest of the agglomerations was above 0.75. Zhejiang and Guangdong Provinces had decreasing efficiencies of scale, and their universities should consider adjusting their methods of management and optimize the scale to match the rates of growth in the output of scientific research with the investment in it. Tianjin, Anhui, Sichuan, and Heilongjiang joined the list of effective provinces in 2020.

### Comparative analysis of efficiency of scientific research in universities in Chengdu–Chongqing economic circle

The efficiency of scientific research in colleges and universities in the Chengdu-Chongqing economic circle has been affected by differences among them. Too large or too small a gap is not conducive to improving the overall efficiency of scientific research of the region. Based on this observation, scholars have proposed a classification standard for universities. According to the new classification standard, the composition of the university is: category and type. It is an improvement over the previous, fixed, model of university classification, and more realistically represents the status of Chinese universities in terms of the scale of scientific research and the subject ratio. We selected research universities in the Chengdu-Chongqing economic circle as a sample for further research.

#### Descriptive statistics

We used "Chinese Universities’ Evaluation " to select 18 research universities in the Chengdu-Chongqing economic circle [35]. 16 research universities were finally used due to lack of data for two of them. We then constructed panel data for them from 2014 to 2018. The lag period was two years. Thus, for example, the data from 2012 to 2014 represented input data from 2012 and output data from 2014.

The annual averages values of various input and output indicators for the 16 research universities are shown in Table 5. It is clear that the size of the input indicators of research-oriented universities in the Chengdu-Chongqing economic circle increased year by year. The average number of senior teaching and scientific research personnel increased from 723 in 2012 to 880 in 2016, an increase of 21.72%, with an average annual growth rate of 5.43%. The average annual growth in the number of full-time equivalent of research and development personnel was 5.59%, the average annual average growth in scientific research funds was 6.89%, and the average annual growth in the number of projects was 9.63%. The total number of subjects taught at research universities in the Chengdu-Chongqing economic circle has grown rapidly, and a large amount of scientific research funds has been allocated to them. By contrast, the growth in the equivalent of senior teaching and research personnel and full-time personnel for research and development has been slow. Therefore, there is a mismatch among the subjects taught, funding, and human input.

**Table 5:** Annual average values of input and output indicators of scientific research by universities in the Chengdu-Chongqing economic circle.

	Tertiary indicator	Year				
		2012–2014	2013–2015	2014–2016	2015–2017	2016–2018
Input	Senior teaching and research personnel (person)	722.56	750.44	800.81	812.25	879.69
	Full-time staff equivalent for research and development (person/year)	698.19	725.75	776.31	782.69	854.31
	Research funds allocated for the year (thousands of yuan)	324759	335679.69	353810	377274.63	414264.25
	Cumulative number of subjects (items)	1253.13	1335.44	1443.19	1589.94	1735.88
Output	published works (department)	6.69	9.94	12.38	12.19	17.50
	academic paper (articles)	2155.81	2176.94	2345.94	2448.94	2535.13
	Actual income from technology transfer (thousands of yuan)	6540.75	5584.13	10992.38	16095.63	13969.50
	Achievement awards (items)	10.56	10.13	11.56	10.50	13.06

Source: “Compilation of Science and Technology Statistics of Higher Education Institutions,” 2012–2018.

The output indicators show that research-oriented universities in the Chengdu-Chongqing economic circle have experienced growth, but only the number of academic papers published has increased year by year (by 17.60%). The number of monographs published decreased slightly in 2017, the income from technology transfer decreased in 2015 and 2018, and the number of achievement awards increased by 23.67% overall, but decreased in 2015 and 2017. This indicates that the university’s output has fluctuated.

#### Assessing the comprehensive efficiency of research universities in Chengdu–Chongqing economic circle

We used DEAP2.1 software to evaluate the input and output efficiency of scientific research of research-oriented universities in Chengdu-Chongqing economic circle. A comparative analysis of their efficiency from 2014 to 2018 is shown in Table 6.

**Table 6:** Efficiency of research-oriented universities in Chengdu–Chongqing economic circle from 2014 to 2018.

Year	Overall Efficiency	Pure Technical Efficiency	Scale Efficiency
	Mean	Mean	Mean
2012–2014	0.525	0.623	0.843
2013–2015	0.647	0.771	0.839
2014–2016	0.748	0.767	0.975
2015–2017	0.781	0.829	0.942
2016–2018	0.854	0.937	0.914
Yearly average	0.711	0.785	0.903

**Fig 1:**
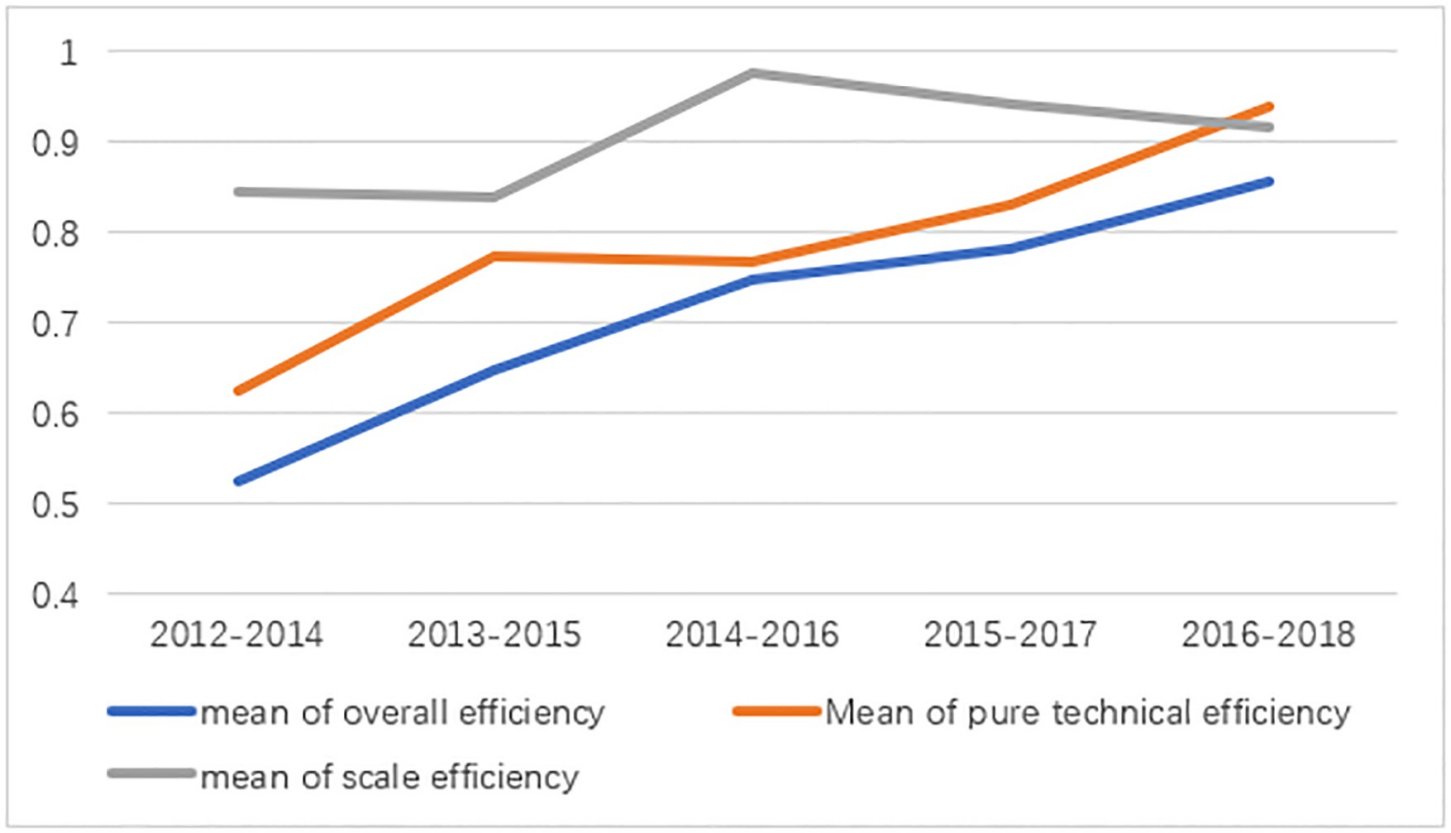
Efficiency of research-oriented universities in Chengdu–Chongqing economic circle from 2014 to 2018. Source: "Compilation of Science and Technology Statistics of Higher Education Institutions” in 2012–2018, compiled in DEAP2.1 software.

Table 6 shows the time series changes of the comprehensive efficiency, pure technical efficiency and scale efficiency of universities in the Chengdu-Chongqing economic circle from 2014 to 2018. The average value of each university continued to increase year by year, but some were still below 0.7 (Fig 1). This shows that there is still considerable room for improvement. The variation in the efficiency of scale was smaller than that in pure technical efficiency, and was close to one. Since overall efficiency = pure technical efficiency * efficiency of scale, pure technical efficiency largely determined comprehensive efficiency in this case. Before 2018, the scale efficiency was always larger than the pure technical efficiency, indicating that the improvement of the former was the main reason for the early growth of the efficiency of universities in the economic circle. However, the scale efficiency in 2018 decreased slightly and was surpassed by pure technical efficiency, indicating that the impact of scale factors on overall efficiency has weakened.

We used the above to examine the similarities and shortcomings among research universities within the Chengdu-Chongqing economic circle in 2018. The results are shown in Table 7.

**Table 7:** Research efficiency of research universities in Chengdu–Chongqing economic circle in 2018.

University	Overall Efficiency	Pure Technical Efficiency	Scale Efficiency	
Chongqing University	0.944	1.000	0.944	drs
Southwest University	0.532	0.566	0.941	drs
Sichuan University	0.940	1.000	0.940	drs
University of Electronic Science and Technology of China	0.804	1.000	0.804	drs
Chongqing University of Posts and Telecommunications	0.748	0.778	0.961	irs
Chongqing Jiaotong University	1.000	1.000	1.000	-
Chongqing Normal University	1.000	1.000	1.000	-
Chongqing University of Technology	1.000	1.000	1.000	-
Chongqing Technology and Business University	1.000	1.000	1.000	-
Chengdu University of Technology	0.933	0.951	0.980	irs
Southwest University of Science and Technology	0.691	0.704	0.981	irs
Southwest Medical University	1.000	1.000	1.000	-
Chengdu University of Traditional Chinese Medicine	1.000	1.000	1.000	-
Sichuan Normal University	0.618	1.000	0.618	irs
West China Normal University	0.661	0.998	0.663	irs
Southwest University for Nationalities	0.796	1.000	0.796	irs
Mean	0.854	0.937	0.914	

“Irs” means increasing returns to scale; “drs” means diminishing returns to scale.

Source: DEAP2.1 software.

The average research efficiency of universities in the Chengdu-Chongqing economic circle was 0.854, which is satisfactory. Among the 16 research-oriented universities, six had a comprehensive efficiency of one. The mean values of pure technology and the efficiency of scale were 0.937 and 0.914, respectively. Four universities were in a state of diminishing returns to scale. If they continue to increase investment in research, the marginal benefits of scale will be lower than their marginal costs.

Six universities-Chongqing Jiaotong University, Chongqing Normal University, Chongqing University of Technology, Chongqing Technology and Business University, Southwest Medical University and Chengdu University of Traditional Chinese Medicine-had been able to effectively allocate their resources for scientific research. rom a purely technical efficiency perspective, many universities reached an effective state of DEA. This shows that their innovative achievements, research management and performance assessment methods are better than before. The other five universities were in a non-DEA-effective state. Their research management efficiency is relatively low. Given that the efficiency of scale = comprehensive efficiency/pure technical efficiency, the scale and pure technical efficiency of Southwest University were both less than one. Its pure technical efficiency was 0.566, which is lower than its scale efficiency of 0.941. This indicates that its lack of effectiveness according to DEA was mainly affected by its pure technical efficiency. Pure technical efficiency thus reflects the comprehensive efficiency of the university as well as its comprehensive ability of scientific research. Chongqing University, Southwest University, Sichuan University, and University of Electronic Science and Technology of China were in a state of decreasing efficiency of scale. They should consider adjusting their management methods and optimizing their scale to match the growth in their output with the investment in scientific research.

#### Projection analysis of non-DEA-effective research universities

To analyze the efficiency of scientific research by various research universities in the Chengdu-Chongqing economic circle, we conducted a predictive analysis of the non-DEA-effective universities in 2018. We calculated the slack in the variables of input and output of these universities to obtain the redundancy of inputs and deficiencies of outputs, and then adjusted the input and output to make the efficiency of scientific research DEA effective. The predictive analysis was performed using DEAP 2.1, and the results are shown in Table 8.

**Table 8:** Predictive analysis of non-DEA-effective universities.

Universities	Deficient Output				Redundant Input			
	Published works (department)	Academic papers (articles)	Actual income from technology transfer (thousands of yuan)	Achievement awards (items)	Senior teaching and research personnel (person)	Full-time staff equivalent for research and development (person/year)	Research funds allocated for the year (thousands of yuan)	Cumulative number of subjects (items)
Southwest University	9.173	0	7997.107	15.037	0	6.466	0	786.943
Chongqing University of Posts and Telecommunications	0	0	0	0.059	0	161.803	36553.756	0
Chengdu University of Technology	3.009	0	15430.561	2.896	0	0	89659.875	375.486
Southwest University of Science and Technology	4.539	0	9928.182	8.969	0	0	0	103.355
West China Normal University	0.679	0	0	0.202	4.225	69.28	0	29.841
Mean	1.087	0	2084.741	1.698	0.264	14.847	7888.352	80.977

Source: Arranged according to the results of DEAP2.1 software.

Judging from the redundancy values of their input elements, five research-oriented universities mainly focused on the equivalent of full-time staff for research and development, the number of projects, and eliminating redundancy in investment in scientific research funds allocated. For example, Chongqing University of Posts and Telecommunications mainly has redundancies of 161.803 people per year and 36553.756 thousand RMB in the equivalent of full-time staff in research and development and the investment in scientific research funds allocated in the year. Universities mainly focused on insufficient output in terms of the number of published monographs, income from technology transfer, and the number of awards for achievements, while their total output of academic papers was high. For example, Southwest University needs to increase the number of published monographs by 9.173, income from technology transfer by 8 million RMB, and the number of awards for achievements by 15.037 to become DEA effective.

This shows that the overall efficiency of scientific research in the above five research-oriented universities was poor. Their non-DEA efficiency were mainly due to the over-input of research personnel and research funds. They need to improve the talent assessment mechanism, strengthen the scientific research management system, and allocate resources rationally according to the differences between their input and output (Table 9).

**Table 9:** Target values of input and output of scientific research in non-DEA-effective universities.

Universities	Output Target Value				Input Target Value			
	Published works (department)	Academic papers (articles)	Actual income from technology transfer (thousands of yuan)	Achievement awards (items)	Senior teaching and research personnel (person)	Full-time staff equivalent for research and development (person/year)	Research funds allocated for the year (thousands of yuan)	Cumulative number of subjects (items)
Southwest University	25.173	2709	8577.107	21.037	686.283	824.09	248654.094	1296.801
Chongqing University of Posts and Telecommunications	13	1235	18305	8.059	422.394	232.588	130868.455	497.072
Chengdu University of Technology	14.009	1842	20530.561	14.896	444.311	318.724	152194.636	656.799
Southwest University of Science and Technology	15.539	1574	10048.182	13.969	432.529	407.169	88657.848	663.08
West China Normal University	3.679	620	0	2.202	283.180	208.145	42823.345	383.304

Source: Arranged according to the running results of DEAP2.1 software.

## Conclusions

This study analyzes the static and dynamic efficiency of research inputs and outputs of universities in 10 urban agglomerations and the Chengdu-Chongqing economic circle in China through DEA-BCC model and Malmquist index. For example, based on the existing literature studies and expert interviews, this study supplemented the input-output indicator system. We selected human resources, research funds, and the number of projects as input indicators, and selected monographs and papers, social benefits, and award-winning achievements as output indicators. Then we used this index system to analyze university research input and output, and reported new insights. Based on the analysis, we draw the following conclusions:

We conducted a comparative analysis of the research efficiency of universities in Chengdu-Chongqing and other urban agglomerations in China showed that in 2020, the average efficiency of scientific research, scale, and comprehensive efficiency of universities increased by 0.015, 0.002, and 0.017, compared with those in 2016. The efficiency of scientific research has generally improved, but only a few urban agglomerations reached the effective level of DEA, and the efficiency gap between them is significant. Although the number of urban agglomerations that have achieved DEA effectiveness in research continues to increase, the level of innovation still needs to be improved, especially in the central and western urban agglomerations. In 2020, the efficiency of scale of colleges and universities in various urban agglomerations increased by 0.2% compared to 2016. The urban agglomerations that achieved effective DEA were Chengdu–Chongqing, Zhongyuan, Harbin-Changzhou, and Guanzhong, and the scale efficiency of the other urban agglomerations was also above 0.75.

From the input indicators, it is clear that the indicators of universities in the Chengdu-Chongqing economic circle increased year by year. The average annual growth rates of the personnel equivalent, senior teaching and scientific research personnel, total funds for scientific research, and total number of projects were 5.59%, 5.43%, 6.89%, and 9.63% respectively. The total number of research projects has grown rapidly and the amount of funding for scientific research is large. There may be a mismatch among the subjects taught, funding, and human input.

We examined the scientific research efficiency and decomposition efficiency of research-oriented universities in the Chengdu-Chongqing economic circle from 2014 to 2018 (the latest year for which data were available). The efficiency of scientific research appears to have significant room for improvement. The improvement in the efficiency of scale before 2018 was the main reason for the early growth in the overall efficiency of research-oriented universities. However, the impact of scale on comprehensive efficiency weakened in 2018. The average efficiency of universities in the Chengdu-Chongqing economic circle was 0.854. Among the 16 research-oriented universities considered in the area, the comprehensive efficiency of six reached one. Four universities were in a state of diminishing returns to scale. From the perspective of pure technical efficiency, five universities were in a non-DEA-effective state, indicating that their research management is less efficient. According to the scale efficiency is equal to the comprehensive efficiency divided by the pure technical efficiency, since the scale and pure technical efficiency of Southwest University are lower than 1 at the same time, and the pure technical efficiency is less than the scale efficiency, it shows that its non-DEA effectiveness is mainly affected by the pure technical efficiency. Therefore, pure technical efficiency reflects the comprehensive efficiency of the university to a large extent, and further reflects the comprehensive scientific research ability of the university.

A predictive analysis of the non-DEA-effective universities in 2018 showed that in the context of the redundancy of input elements, five institutions focused on the equivalent of full-time R&D personnel, the number of projects, and the funds allocated for scientific research. In the context of insufficiency of output, the universities focused on the number of published monographs, income from technology transfer, and the number of awards for achievements. The overall efficiency of scientific research of the five research universities (Southwest University, Chongqing University of Posts and Telecommunications, Chengdu University of Technology, Southwest University of Science and Technology, West China Normal University) was low. Their non-DEA effectiveness was due to excessive investment in scientific research personnel.

## Discussion

This paper constructs an evaluation index system of scientific research efficiency of universities. Using the DEA method analyzes the input and output of scientific research efficiency in 10 urban agglomerations in China and universities in the Chengdu-Chongqing economic circle. We calculated and compared their scientific research input-output efficiency, and then conducted a comparative analysis of the differences in scientific research efficiency among the research type sample universities within Chengdu-Chongqing economic circle. Through the above efficiency evaluation and difference analysis, relevant universities can explore more ways to improve their own scientific research efficiency from connotation, management, system and other aspects in the future. Therefore, we propose the following suggestions:

The study shows that the scientific research efficiency of universities in Chengdu-Chongqing economic circle still has a large space for improvement, and the pure technical efficiency largely determines the comprehensive efficiency. Before 2018, the growth of comprehensive efficiency of universities mainly depended on the improvement of scale efficiency, while the influence of scale factor on comprehensive efficiency was weakened after 2018. Research universities in the Chengdu–Chongqing region should engage in scientific research according to their situation, subdivide or reorganize institutions of research, integrate research into regional economic development, expand its autonomy, and strengthen protections for intellectual property. A scientific research evaluation system with quality and efficiency at its core is needed. They can improve their pure technical efficiency only by fully exploiting their technical advantages, enhancing the awareness of development, and strengthening economic cooperation with surrounding areas.

Through a comparative analysis of the scientific research efficiency of universities in some urban agglomerations, it is found that only a few urban agglomerations are in the DEA effective state, and the gap between urban agglomerations is relatively significant. The scientific research activities of universities in urban agglomerations are not isolated from the world, but closely depend on interactions with it. Their efficiency gradually converges over time. To strengthen competition and cooperation among various cities within the urban agglomeration, large and central cities should play an exemplary role by assisting surrounding, smaller cities in their development. The latter should in turn learn from the experience of the former to improve their level of economic development. Universities in urban agglomerations, especially different types of universities, should promote information exchange and strengthen the flow of research talent into different disciplines.

Through the comparative analysis of the research sample universities in the Chengdu-Chongqing Economic Circle, it can be seen that the total number of research universities has increased rapidly, and the amount of allocated scientific research funds is large. Compared with that, the equivalent growth rate of teaching and research personnel with senior professional titles, research and development full-time personnel is relatively slow. Universities in the Chengdu-Chongqing region must attend to the cultivation of local talent for scientific research and innovation. Moreover, greater emphasis should be placed on the appropriate allocation of scientific research personnel. The government should gradually establish a performance appropriation system based on the efficiency of scientific research by universities, and allocate higher-quality resources to more efficient universities. Furthermore, the relevant education department should give more autonomy to universities in terms of personnel, and increase the structure of apportioning of teachers, and senior personnel for research and full-time R&D personnel. Finally, research-oriented universities should consider deploying more scientific research assistants to improve the efficiency of handling routine research-related affairs and to enable researchers to better focus on their primary tasks.

The research results of this paper provide reference suggestions for the research on scientific research efficiency of universities in urban agglomeration and Chengdu-Chongqing economic circle, but there are also some limitations for further research in the future. These factors include: (1) This paper selected the usual BCC model and Malmquist index of DEA method, how to further improve the scientific nature of the model is the future research direction. (2) It is necessary to increase the reading of literature, increase the sample size of indicator selection, and build a more reasonable and objective evaluation index of university scientific research efficiency. (3) At the same time, the depth of data analysis is not enough. It should be extended to a wider range of applications.

## Supporting information

S1 Appendix Supporting information (including supporting tables and data source on-line). (DOCX)
