# Supplementary material for: Scientific research input and output efficiency evaluation of universities in Chengdu–Chongqing economic circle based on data envelopment analysis
Source: PLoS One. 2023 Jul 7;18(7):e0287692. doi: 10.1371/journal.pone.0287692 (PMC10328349; doi:10.1371/journal.pone.0287692)
Supplement: S1 Appendix — (DOCX) [file pone.0287692.s001.docx]

**Supporting information**

Table S1 Senior teaching and research personnel in 2016–2020

| Senior teaching and research personnel (persons) | | | | | | | | | | | | | | | |
| --- | --- | --- | --- | --- | --- | --- | --- | --- | --- | --- | --- | --- | --- | --- | --- |
|  | Jiangsu | Beijing | Guangdong | Shandong | Hubei | Shanghai | Henan | Shanxi | Zhejiang | Liaoning | Hunan | Hebei | Sichuan | Heilongjiang | Chongqing |
| 2020 | 36538 | 32271 | 30492 | 28881 | 25397 | 22780 | 21422 | 20987 | 20394 | 19978 | 18968 | 18719 | 18623 | 17310 | 10301 |
| 2019 | 32863 | 30614 | 27538 | 25542 | 23812 | 21535 | 17909 | 20105 | 19297 | 19658 | 18130 | 18061 | 19453 | 16558 | 9146 |
| 2018 | 32736 | 29167 | 25930 | 24218 | 22989 | 20108 | 17073 | 18839 | 18585 | 19093 | 17006 | 16822 | 18453 | 16383 | 9090 |
| 2017 | 31393 | 28380 | 22988 | 21355 | 22161 | 18399 | 13275 | 17301 | 17470 | 18271 | 15876 | 15859 | 16920 | 15687 | 8393 |
| 2016 | 29749 | 27169 | 20959 | 21461 | 20436 | 17063 | 12877 | 15992 | 16006 | 17549 | 14919 | 13592 | 16026 | 15512 | 8072 |

Table S2 Full-time staff equivalent for research and development in 2016–2020

| Full-time staff equivalent for research and development (person/year) | | | | | | | | | | | | | | | |
| --- | --- | --- | --- | --- | --- | --- | --- | --- | --- | --- | --- | --- | --- | --- | --- |
|  | Beijing | Shanghai | Jiangsu | Guangdong | Shandong | Shanxi | Sichuan | Hubei | Liaoning | Zhejiang | Anhui | Heilongjiang | Hunan | Jilin | Chongqing |
| 2020 | 60492 | 38288 | 38085 | 32886 | 28090 | 25675 | 23846 | 23134 | 22768 | 21220 | 19891 | 19709 | 19573 | 18164 | 9217 |
| 2019 | 34417 | 28274 | 23565 | 24778 | 22007 | 12681 | 17460 | 13597 | 18642 | 14932 | 13194 | 14130 | 13398 | 14137 | 7488 |
| 2018 | 32749 | 24078 | 23493 | 22583 | 20485 | 11225 | 16218 | 13605 | 16661 | 14447 | 11552 | 12674 | 14314 | 13474 | 7309 |
| 2017 | 29583 | 23266 | 23562 | 20598 | 18810 | 10210 | 14876 | 12736 | 16421 | 12578 | 11681 | 13864 | 12786 | 13510 | 6635 |
| 2016 | 31447 | 21874 | 20427 | 19947 | 18996 | 9636 | 13893 | 12857 | 15742 | 11657 | 12441 | 14577 | 11806 | 14257 | 5745 |
|  |  |  |  |  |  |  |  |  |  |  |  |  |  |  |  |

Table S3 Research funds allocated per year in 2016–2020

| Research funds allocated for the year (thousands of yuan) | | | | | | | | | | | | | | | |
| --- | --- | --- | --- | --- | --- | --- | --- | --- | --- | --- | --- | --- | --- | --- | --- |
|  | Beijing | Jiangsu | Shanghai | Guangdong | Zhejiang | Shanxi | Hubei | Shandong | Sichuan | Hunan | Anhui | Heilongjiang | Liaoning | Chongqing | Tianjin |
| 2020 | 35476706 | 23589298 | 21186409 | 20375865 | 14876719 | 13772011 | 13696661 | 13129516 | 9094398 | 8070556 | 7664355 | 6748023 | 6120597 | 5155519 | 5153482 |
| 2019 | 30000805 | 19555358 | 17614800 | 17520700 | 11106366 | 10867955 | 11808201 | 9647078 | 7597508 | 6067594 | 6820689 | 6464625 | 5739030 | 4107555 | 5018605 |
| 2018 | 25368895 | 17356174 | 14986233 | 15842487 | 9162636 | 9243642 | 10012318 | 7987797 | 6330685 | 4919778 | 6853140 | 5766399 | 4798874 | 3606782 | 4145777 |
| 2017 | 23109679 | 15996884 | 13683902 | 13552865 | 8276940 | 7896989 | 8673432 | 5613099 | 5590918 | 4061834 | 4719601 | 4984151 | 5433676 | 3326692 | 3774561 |
| 2016 | 22194989 | 13955124 | 11543879 | 9024679 | 7369156 | 7003847 | 7507086 | 4783032 | 5243434 | 3580164 | 4516390 | 4746769 | 5379566 | 2310431 | 3483898 |

Table S4 Academic papers in 2016–2020

| Academic papers (articles) | | | | | | | | | | | | | | | |
| --- | --- | --- | --- | --- | --- | --- | --- | --- | --- | --- | --- | --- | --- | --- | --- |
|  | Jiangsu | Beijing | Guangdong | Shanghai | Shanxi | Hubei | Sichuan | Shandong | Hunan | Liaoning | Zhejiang | Heilongjiang | Henan | Anhui | Chongqing |
| 2020 | 110356 | 99197 | 80631 | 79040 | 62171 | 60395 | 59355 | 57978 | 44780 | 42410 | 40016 | 38708 | 35418 | 30404 | 26162 |
| 2019 | 101418 | 97999 | 77876 | 71225 | 55886 | 60692 | 59437 | 56744 | 41170 | 41320 | 38371 | 34274 | 33515 | 27979 | 22338 |
| 2018 | 92944 | 96704 | 62605 | 67020 | 49131 | 59017 | 52419 | 50086 | 37965 | 40134 | 37257 | 31851 | 28366 | 28311 | 22083 |
| 2017 | 86037 | 87213 | 63291 | 61943 | 45510 | 56487 | 51419 | 41366 | 34756 | 40882 | 33724 | 29247 | 31070 | 27359 | 21629 |
| 2016 | 86525 | 85613 | 49405 | 59957 | 43013 | 52584 | 50140 | 39667 | 34093 | 35992 | 29246 | 30932 | 31077 | 29429 | 19599 |

Table S5 Published works in 2016–2020

| Published works (department) | | | | | | | | | | | | | | | |
| --- | --- | --- | --- | --- | --- | --- | --- | --- | --- | --- | --- | --- | --- | --- | --- |
|  | Beijing | Henan | Jiangsu | Shandong | Shanghai | Shanxi | Sichuan | Liaoning | Hubei | Guangdong | Zhejiang | Heilongjiang | Hunan | Chongqing | Anhui |
| 2020 | 625 | 451 | 369 | 363 | 347 | 342 | 332 | 319 | 314 | 274 | 197 | 197 | 194 | 186 | 170 |
| 2019 | 636 | 455 | 379 | 282 | 302 | 321 | 387 | 411 | 315 | 302 | 174 | 172 | 233 | 185 | 186 |
| 2018 | 573 | 424 | 461 | 220 | 271 | 260 | 288 | 351 | 323 | 231 | 163 | 247 | 217 | 146 | 131 |
| 2017 | 595 | 315 | 418 | 109 | 252 | 221 | 218 | 297 | 278 | 194 | 135 | 210 | 154 | 115 | 142 |
| 2016 | 509 | 224 | 370 | 173 | 224 | 237 | 231 | 316 | 221 | 171 | 144 | 217 | 113 | 78 | 99 |

Table S6 Actual income from technology transfer in 2016–2020

| Actual income from technology transfer (thousands of yuan) | | | | | | | | | | | | | | | |
| --- | --- | --- | --- | --- | --- | --- | --- | --- | --- | --- | --- | --- | --- | --- | --- |
|  | Jiangsu | Shandong | Shanghai | Guangdong | Sichuan | Shanxi | Liaoning | Zhejiang | Beijing | Hunan | Chongqing | Hubei | Henan | Fujian | Heilongjiang |
| 2020 | 213635 | 156671 | 153039 | 149582 | 123025 | 122341 | 108111 | 103865 | 89017 | 71801 | 60113 | 55540 | 21359 | 18563 | 15811 |
| 2019 | 154090 | 90223 | 343332 | 164148 | 17826 | 112352 | 168830 | 50109 | 529947 | 87072 | 15235 | 60762 | 24695 | 8897 | 7648 |
| 2018 | 188064 | 577123 | 117468 | 26141 | 75794 | 29204 | 134351 | 97505 | 493770 | 14181 | 23077 | 90510 | 23563 | 13687 | 8248 |
| 2017 | 130163 | 15679 | 94863 | 22164 | 25303 | 34037 | 58815 | 89201 | 518696 | 8673 | 4892 | 31392 | 12500 | 94998 | 16413 |
| 2016 | 65853 | 9774 | 35702 | 17050 | 35035 | 24323 | 6917 | 26251 | 284422 | 12247 | 14644 | 29691 | 13725 | 10230 | 4241 |

Table S7 Achievement awards in 2016–2020

| Achievement awards (items) | | | | | | | | | | | | | | | |
| --- | --- | --- | --- | --- | --- | --- | --- | --- | --- | --- | --- | --- | --- | --- | --- |
|  | Jiangsu | Beijing | Hubei | Shanxi | Shanghai | Liaoning | Heilongjiang | Anhui | Zhejiang | Hunan | Henan | Shandong | Sichuan | Guangdong | Chongqing |
| 2020 | 499 | 371 | 360 | 282 | 279 | 258 | 253 | 239 | 237 | 237 | 236 | 229 | 219 | 192 | 131 |
| 2019 | 457 | 396 | 357 | 256 | 295 | 237 | 239 | 137 | 256 | 185 | 184 | 172 | 216 | 140 | 136 |
| 2018 | 447 | 424 | 339 | 243 | 279 | 246 | 222 | 131 | 218 | 175 | 215 | 163 | 252 | 188 | 106 |
| 2017 | 385 | 442 | 326 | 213 | 280 | 341 | 235 | 135 | 224 | 210 | 152 | 131 | 205 | 197 | 88 |
| 2016 | 394 | 502 | 315 | 223 | 323 | 349 | 259 | 148 | 171 | 194 | 143 | 148 | 192 | 196 | 93 |

Table S8 Cumulative number of subjects in 2016–2020

| Cumulative number of subjects (items) | | | | | | | | | | | | | | | |
| --- | --- | --- | --- | --- | --- | --- | --- | --- | --- | --- | --- | --- | --- | --- | --- |
|  | Beijing | Jiangsu | Guangdong | Shanghai | Shanxi | Zhejiang | Hubei | Sichuan | Shandong | Liaoning | Hunan | Fujian | Anhui | Chongqing | Heilongjiang |
| 2020 | 72948 | 65205 | 55592 | 48285 | 43222 | 41857 | 40959 | 39888 | 37994 | 24954 | 29298 | 27331 | 22837 | 19688 | 18547 |
| 2019 | 69706 | 57125 | 55187 | 43655 | 37542 | 38483 | 35676 | 38190 | 32967 | 22743 | 21222 | 22260 | 20037 | 16565 | 15317 |
| 2018 | 63698 | 50899 | 48910 | 38767 | 35337 | 36800 | 30304 | 32893 | 28034 | 20571 | 17684 | 19592 | 18446 | 14750 | 14437 |
| 2017 | 61006 | 48734 | 44184 | 34967 | 33156 | 32389 | 30469 | 29248 | 22830 | 19137 | 17358 | 18104 | 17725 | 13819 | 13787 |
| 2016 | 61907 | 42988 | 37885 | 35115 | 30429 | 28514 | 30489 | 25449 | 21929 | 17644 | 16830 | 15229 | 17435 | 12571 | 15227 |

**Source: "Compilation of Science and Technology Statistics in Higher Education Institutions" in 2016–2020 (**[**http://www.moe.gov.cn/s78/A16/**](http://www.moe.gov.cn/s78/A16/)**).**

The above data are compiled by the research team based on the initial data. The initial source of all relevant data used in this study is the public data on the website of the Ministry of Education of China (address: http://www.moe.gov.cn/s78/A16/A16_tjdc/), the document “Compilation of Science and Technology Statistics in Higher Education Institutions” .

The above is about our reply to all the questions this time. If you have any other questions or doubts, we will try our best to answer. Special thanks to you for your good comments.
